# Performance of an Automated Zika IgG Immunoassay in the Detection of Zika IgG Specific Antibodies—A Validation Approach in Samples from Prevalence Areas and Non-Endemic Countries

**DOI:** 10.3390/tropicalmed5020097

**Published:** 2020-06-08

**Authors:** Tina Laengin, Stephanie Augenstein, Elke Stadlbauer, Heike Girgnhuber, Mario Gloeck, Alexander Riedel

**Affiliations:** Centralised and Point of Care Solutions, Roche Diagnostics GmbH, D-82377 Penzberg, Germany; tina.laengin@roche.com (T.L.); stephanie.lepa@roche.com (S.A.); elke.stadlbauer@roche.com (E.S.); heike.girgnhuber@roche.com (H.G.); mario.gloeck@roche.com (M.G.)

**Keywords:** Zika virus, serology, diagnosis, immunoglobulin, sensitivity, specificity

## Abstract

The diagnosis of Zika virus infection is complicated and includes testing for nucleic acids and IgM and IgG antibodies, depending on the stage of infection. Zika IgG is an important marker of infection after the acute stage; however, IgG assays can lack specificity due to the similarities between Zika and other flaviviruses. In this study, the diagnostic sensitivity and specificity of the Elecsys^®^ Zika IgG assay were assessed in 496 samples from Zika endemic regions, and specificity only was assessed in 1685 blood screening and diagnostic samples from Zika non-endemic regions. Cross-reactivity was also assessed against a panel of 202 potentially cross-reacting samples. The performance of the Elecsys^®^ Zika IgG assay was compared with the anti-Zika virus ELISA IgG. In the samples from the Zika endemic regions, the Elecsys^®^ Zika IgG assay had 92.88% (95% confidence interval 89.42–95.48) sensitivity and 100% specificity and in the samples from Europe the Elecsys^®^ Zika IgG assay specificity was ≥99.62%. The Elecsys^®^ Zika IgG assay was highly specific in samples from both prevalent and non-endemic regions.

## 1. Introduction

The Zika virus (ZIKV) is a mosquito-borne flavivirus that was first isolated in 1947 from a febrile rhesus macaque in the Zika forest of Uganda through a yellow fever surveillance network in the area [1]. The following year, the ZIKV was isolated from *Aedes* mosquitoes from the same forest [2]. The first human case of infection with ZIKV occurred in Uganda in 1962–3 [3]. Recently, the virus has become more widely known due to a series of epidemics starting in Micronesia in 2007 and the eventual emergence of ZIKV in Brazil in 2014 [4]. Since then, the ZIKV has spread considerably in the Americas and has also been reported in Europe [4,5].

Transmission of the virus to humans is primarily through the bite of an infected *Aedes* mosquito species, although transmission may also occur through several non-vector-borne routes, including pre- and peri-natal transmission, sexual intercourse, and blood transfusions [6,7,8]. The increasing worldwide presence of the *Aedes* mosquito species may lead to the emergence of new ZIKV epidemics in urban areas [9].

ZIKV infection is asymptomatic in an estimated 80% of cases [10,11,12]. When symptomatic, ZIKV infection usually presents with non-specific influenza-like symptoms, including rash, fever, arthralgia, myalgia, headache, and conjunctivitis, typically lasting 3–6 days [10,12]. Infections may be clinically difficult to distinguish from diseases caused by other arboviruses including Dengue virus (DENV), Chikungunya, and West Nile virus [9].

Complications of ZIKV infection include Guillain–Barré syndrome, a neurologic disorder that can lead to paralysis and death [9]. Pre-natal ZIKV infection can cause serious neurologic sequelae including, but not limited to, microcephaly, ventriculomegaly, intracranial calcifications, and ocular abnormalities [8].

Laboratory evidence of ZIKV infection can be obtained by testing clinical samples (biofluids and tissue) for viral nucleic acid or virus-specific IgM and IgG antibodies [12]. Serologic testing is recommended in individuals if the specimen is collected more than 1 week after the onset of symptoms [13]. Due to the clinical manifestations and the associated consequences, diagnostic requests in those countries at the highest risk of a ZIKV outbreak are forecast to increase substantially [14]. The ZIKV shares a considerable degree of structural homology with other flaviviruses [15,16]. Serology-based diagnosis has historically posed a challenge due to the well-known problem of potential cross-reactivity with antibodies produced, particularly against other flaviviruses including DENV [12].

Currently, there are neither vaccines to prevent Zika nor effective drugs for the treatment of already infected patients [17]. Improvements in the surveillance and monitoring of Zika infection would support the efforts to combat this viral infection [17].

Due to the similarity of ZIKV to other viruses, the Elecsys^®^ Zika IgG assay was developed as a highly specific assay to limit cross-reaction and reduce the occurrence of false-positive results. The objective of this study was to evaluate the specificity of the Elecsys^®^ Zika IgG assay, a qualitative one-step double-antigen sandwich (DAGS) immunoassay using recombinant ZIKV antigens, designed for the in vitro detection of anti-Zika IgG antibodies in human serum and plasma, using samples from: ZIKV prevalence areas, blood donors from Europe, pregnant women from Europe, and samples from other viral, bacterial, and parasitic infections.

## 2. Methods

### 2.1. Study Design

This was an analytical performance evaluation of the Elecsys^®^ Zika IgG assay using the **cobas e** 601 platform. The performance of the Elecsys^®^ Zika IgG assay was compared with that of the anti-Zika virus ELISA IgG (EUROIMMUN, Lübeck, Germany) [18]. Testing of the Elecsys^®^ Zika IgG was performed at TRIGA-S Scientific Solutions, Habach, Germany. All other testing was performed at Roche Diagnostics GmbH (Penzberg, Germany).

### 2.2. Samples

Frozen serum/plasma specimens from different populations from prevalent and non-endemic regions were used to assess the specificity of the Elecsys^®^ Zika IgG assay. Samples from prevalent areas included patients from Latin America with suspected Zika infection (N = 396) and patients in the Côte d’Ivoire (N = 100). Samples from non-endemic countries included presumed negative samples from Latin America (collected prior to Zika epidemics; N = 94), samples from pregnant women from Europe (N = 500), blood donors from Europe (N = 532), and blood donors from tick-borne encephalitis (TBE) endemic regions with high TBE vaccination coverage (N = 559). All samples were obtained from the following commercial vendors: Medical Research Networx LLC, Biomex AG, Blood-Bank Innsbruck, Teragenix Inc., Biomex AG, TrinaBioreactives AG, Agencia Nacional de Vigilancia Sanitaria, and Roche Molecular Diagnostics, Indianapolis. The study was conducted according to the study protocol provided by Roche Diagnostics and in accordance with the Declaration of Helsinki: patient samples were fully anonymized, leftover samples that were obtained with signed informed consent and were preserved for research use.

For the analytical specificity analysis, samples that were positive for non-Zika infections (N = 202) were also commercially obtained as described above.

### 2.3. Methods and Analyses

**Figure 1:**
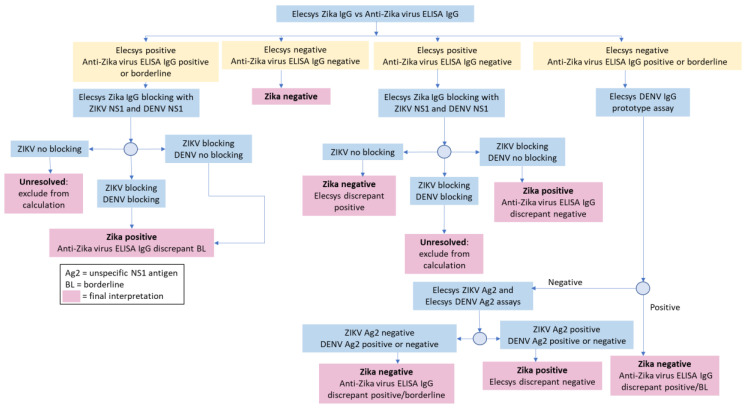
Resolution testing algorithm.

The sensitivity and specificity of the Elecsys^®^ Zika IgG assay were determined using a testing algorithm: the comparator serologic test, followed by further resolution of any discrepant samples using blocking experiments (using Zika/Dengue full length NS1 protein) and/or the *recom*LINE Tropical Fever IgG immunoblot assay (MIKROGEN GmbH, Neuried, Germany; Figure 1) [19]. The testing algorithm provided a surrogate standard for assessing diagnostic performance. Diagnostic specificity, diagnostic sensitivity, and confidence intervals ((CI) 95%, two-sided) were calculated using a validated statistical analysis system (SAS) tool (developed by Biostatistics, Roche Diagnostics GmbH).

## 3. Results

### 3.1. Diagnostic Performance

#### 3.1.1. Zika Endemic Areas

The Elecsys^®^ Zika IgG assay was assessed in a total of 496 samples from ZIKV endemic areas. In these samples, the Elecsys^®^ Zika IgG assay had 100% specificity, correctly determining 151 Zika-negative samples (Table 1). The 99 samples that were determined negative by both the Elecsys^®^ Zika IgG assay and the anti-Zika virus ELISA IgG were not further tested and did not need resolution. In total, 52 samples that were confirmed negative by resolution testing were either reactive or borderline with the anti-Zika virus ELISA IgG which had 65.56% (95% CI, 57.40–73.10) specificity.

To assess the sensitivity of the Elecsys^®^ Zika IgG assay compared with the anti-Zika virus ELISA IgG, the 397 samples that were either concordant positive or discordant were further tested with the resolution algorithm (Figure 1). The Elecsys^®^ Zika IgG assay identified 287 of the 309 positive samples and was 92.88% (95% CI, 89.42–95.48) sensitive. The anti-Zika virus ELISA IgG identified all 309 samples as Zika positive or borderline and was 100% (95% CI, 98.81–100) sensitive. Of the 22 samples that were Elecsys^®^ Zika IgG negative but discrepant, further testing found that 10 were positive for DENV IgG antibodies, 10 were positive for flavivirus IgGs and two samples remained inconclusive.

#### 3.1.2. Zika Non-Endemic Regions

In 1685 routine diagnostic and blood screening samples from Europe, the Elecsys^®^ Zika IgG assay had a specificity of 99.88% (95% CI, 99.57–99.99) (Table 2). The specificity was 100% in most groups and only differed in the general European blood donor group. Specificity was not reduced in patients from the high TBE vaccination status group. Due to the small number of Zika-positive samples in this cohort, it was not appropriate to assess the assay sensitivity.

### 3.2. Analytical Specificity

A total of 202 potentially cross-reacting samples, positive for other “non-Zika” infections, that were Zika-negative using the anti-Zika virus ELISA IgG were all also Zika-negative with the Elecsys^®^ Zika IgG assay (Table 3).

## 4. Discussion

In this study, the specificity of the Elecsys^®^ Zika IgG assay was high in all sample cohorts from endemic and non-endemic areas. This is important as cases of Zika are reported in both scenarios and also in the interest of being able to detect historic infection and to help predict future outbreaks of the disease [20,21]. The Center for Disease Control and Prevention currently advises caution against the potential cross-reactivity of Zika IgG assays [21]; however, in this study we found no cross-reactivity with any of the other infections that were assessed. The Elecsys^®^ Zika IgG assay was also highly specific in patients with high vaccine status for TBE. In the samples from Zika endemic regions, there were no false positives using the Elecsys^®^ Zika IgG assay and only two samples yielded false positives with the Elecsys^®^ Zika IgG assay in this study. Further analysis of the negative Elecsys^®^ Zika IgG discrepant samples from the Zika endemic region found that some were DENV positive or positive for general flavivirus. This suggests that the Elecsys^®^ Zika IgG does not cross-react with DENV, which is a known problem for Zika IgG assays [22,23]. Like the anti-Zika virus ELISA IgG, the Elecsys^®^ Zika IgG assay was designed to target antibodies to the ZIKV NS1 antigen; however, the Elecsys^®^ Zika IgG assay targeted only the immunodominant wing domain in order to avoid cross-reactivity [24]. In addition to the specificity shown in this study, the sensitivity of the Elecsys^®^ Zika IgG was >92% and was similar to that of the anti-Zika virus ELISA IgG. This suggests that the specificity of the Elecsys^®^ Zika IgG assay is not improved by compromising the sensitivity of the assay. This is important as commercially available Zika assays have been shown to have either low sensitivity or specificity in a clinical setting [25,26].

Due to its persistence in the blood, Zika IgG is particularly useful for mapping patterns of infection and predicting future outbreaks and is used by the WHO in reporting and confirming outbreaks [13]. Zika IgG has also been found to be present in high levels in infants born with microcephaly, suggesting it might also be important in assessing maternal–fetal disease transmission [27]. This study demonstrates that the Elecsys^®^ Zika IgG assay is highly specific and is a promising tool for use in monitoring Zika seroprevalence and infection.

## Figures and Tables

**Table 1:** Elecsys^®^ Zika IgG and anti-Zika virus ELISA IgG assay diagnostic sensitivity and specificity in samples from endemic areas.

Cohort	N	Positive or Borderline* Samples after Resolution	Negative Samples after Resolution	Unresolved Samples	Negative Discrepant Samples after Resolution^†^	Sensitivity (95% CI)	Specificity (95% CI)
**Elecsys^®^ Zika IgG**							
Suspected Zika infection from Latin America	396	284	55	36	21	93.11 (89.67–95.69)	100 (93.51–100)
Samples from Côte d’Ivoire	100	3	96	0	1	75.00 (19.41–99.37)	100 (96.23–100)
Total	496	287	151	36	22	92.88 (89.42–95.48)	100 (97.59–100)
**Anti-Zika Virus ELISA IgG**							
Suspected Zika infection from Latin America	396	305	21	36	34	100.00 (98.80–100)	38.18 (25.41–52.27)
Samples from Côte d’Ivoire	100	4	78	0	18	100 (39.76–100)	81.25 (72.00–88.49)
Total	496	309	99	36	52	100 (98.81–100)	65.56 (57.40–73.10)

* Only the anti-Zika virus ELISA IgG had borderline results. ^†^ There were no positive discrepant samples after resolution testing.

**Table 2:** Elecsys^®^ Zika IgG assay diagnostic specificity in samples from non-endemic areas.

Cohort	N	Positive Samples after Resolution	Negative Samples after Resolution	Positive Discrepant Samples after Resolution	Specificity (95% CI)
Samples from Latin America before Zika epidemics	94	2	92	0	100 (96.07–100)
Pregnant women in Europe	500	0	500	0	100 (99.26–100)
European blood donors	532	4	526	2	99.62 (98.64–99.95)
European blood donors with high TBE vaccination status	559	0	559	0	100 (99.34–100)
Total	1685	6	1677	2	99.88 (99.57–99.99)

**Table 3:** Assessment of the Elecsys^®^ Zika IgG assay in potentially cross-reacting samples.

Pathogen	N	Non-Reactive	Reactive
DENV	30	30	0
Cytomegalovirus	12	12	0
Epstein–Barr virus	11	11	0
Herpes simplex virus	10	10	0
Hepatitis A virus	8	8	0
Rubella virus	12	12	0
Hepatitis B virus	11	11	0
Hepatitis C virus	12	12	0
*Plasmodium falciparum/vivax* (malaria)	11	11	0
*Treponema pallidium* (syphilis)	12	12	0
Varicella zoster virus	12	12	0
TBE virus	20	20	0
Yellow fever virus	15	15	0
Japanese encephalitis virus	3	3	0
Antinuclear antibodies	12	12	0
Rheumatoid factor	11	11	0
